# A study to investigate the incidence of early satiety in patients with advanced cancer.

**DOI:** 10.1038/bjc.1992.98

**Published:** 1992-03

**Authors:** P. J. Armes, H. J. Plant, A. Allbright, T. Silverstone, M. L. Slevin

**Affiliations:** Imperial Cancer Research Fund, Department of Medical Oncology, St Bartholomew's Hospital, London.


Br. J. Cancer (1992), 65, 481-484 © Macmillan Press Ltd., 1992

SHORT COMMUNICATION

### A study to investigate the incidence of early satiety in patients with advanced cancer

P.J. Armes[1], H.J. Plant[1], A. Allbright[1], T. Silverstone[2], & M.L. Slevin[1]

[1]*Imperial Cancer Research Fund, Department of Medical Oncology, St Bartholomew's and Homerton Hospitals, London;* [2]*Department of Psychological Medicine, St Bartholomews's and Homerton Hospital, London.*

Cancer cachexia is debilitating and distressing, not only for the patient, but for the family and friends who feel helpless to stop their relative 'dissolving before their eyes'. Eating is normally a pleasurable and sociable occasion, which contributes to a sense of well-being. The normal physiology of hunger, appetite and satiety continue to be conjectural. What is known is that the hypothalamus plays a major facilitatory and inhibitory role in hunger and satiety. Feedback from plasma glucose, free fatty acids, glycerol, amino acids, peripheral metabolic state and signals from the gastrointestinal tract ensure the fine tuning of calorie intake, distribution, disposal and storage (Bray & Campfield 1975). Mayer (1953, 1955, 1957) suggested that blood glucose may be the link between the supply of nutrients and the hypothalamus. A 'normal' hunger is a result of physiological changes in the state of the body's stores of energy and is inhibited by fullness, a sensation arising from the alimentary canal (Davidson *et al.*, 1979). The desire to eat can be seen as a continuum with appetite at one end and satiety at the other, with the change occurring gradually as the meal progresses (Silverstone & Goodall, 1986).

Cancer cachexia is viewed as a complex syndrome, the aetiology of which is still uncertain and is often only reversed when the malignancy is treated effectively (Theologides, 1977). It occurs in one-half to two-thirds of patients with cancer and has long been recognised as a frequent cause of death in the cancer patient (Warren, 1932). The symptoms of cancer cachexia are anorexia, early satiety, loss of body protein and fat, anaemia and marked asthenia. In addition, there is an increase in the basal metabolic rate and a corresponding increase in energy expenditure, whilst there is frequently a decreased energy intake (Theologides, 1979).

Metabolic factors play a role in many patients and may include: altered carbohydrate and protein metabolism (Holroyde & Reichard, 1981; Young *et al.*, 1979), selective use and redistribution of nutrients by tumour cells (Theologides, 1979), alterations of total lipid mobilisation resulting in elevated free fatty acids (Knox *et al.*, 1983) and abnormal synthesis of peptides which may disrupt normal enzyme activity (Theologides, 1972; Young, 1977). Cancer patients commonly demonstrate disturbances of glucose metabolism, Glicksman & Rawson (1956) described this as a diabetic glucose tolerance. This is characterised by reduced insulin sensitivity (Lundholm *et al.*, 1978), increased glucose turnover (Waterhouse, 1974; Holroyde *et al.*, 1975), altered insulin production (Kisner *et al.*, 1978), reduction in the disappearance rate of i.v. glucose (Marks & Bishop, 1957), increased gluconeogenesis (Holroyde *et al.*, 1975), and an increase in anaerobic glucose consumption (Warbur 1956; Waterhouse, 1974) which leads to an increased level of lactate and so to an increased rate of Cori cycling. It has been postulated that these changes in glucose metabolism may lead to anorexia, satiety, nausea and weight loss (De Wys, 1979).

A large component of cancer cachexia is reduced food intake. Theologides (1979) has suggested that in addition to anorexia, early satiety may be a major factor contributing to reduced food intake. Early satiety is the desire to eat associated with an inability to eat more than a few mouthfuls, as illustrated in a study carried out by Theologides (1976) where 10 patients denied anorexia but complained of easy filling, despite a good initial appetite. This is distinct from anorexia where there is a reduced desire to eat. Lindsey & Piper (1985) report that early satiety and nausea were experienced most frequently by those patients who reported any appetite-related symptoms. It has been suggested that early satiety is seen with increasing frequency in those with advanced cancer, especially following chemotherapy (Theologides, 1976), however Neilsen *et al.*, (1980) demonstrated no significant differences in early satiety between patients receiving cytotoxic chemotherapy and those not. This study also showed that there were no significant differences in tumour type between the numbers of patients with and without early satiety. When patients were divided by the presence or not of taste aversions it was found that 80% of those with taste aversions ($n = 56$) experienced early satiety compared with 49% of those without taste aversion ($n = 77$). In addition, those patients experiencing early satiety found the odours of pork, ham, and coffee significantly less pleasant than those without early satiety (Neilsen *et al.*, 1980). It has been noted that anorexia and early satiety appear to worsen during the day (Knox *et al.*, 1983; Grant, 1986).

The pathogenesis of early satiety on the whole is unclear, however some theories have been proposed. It may be caused by direct encroachment of the tumour on the gastrointestinal tract (Grant, 1986). Theologides (1974) has hypothesised that early satiety is a result of satiety signals sent by oropharyngeal receptors. Others have suggested that it may be due to atrophic changes in the mucosa and muscles of the stomach, and a reduction in the secretion or activity of gastrointestinal enzymes which may lead to delayed gastric emptying, slowing in peristalsis, and sustained stimulation of receptors in the gastrointestinal tract, and, ultimately, in early satiety and a decreased stimulation of appetite (Knox *et al.*, 1983; Grant, 1986; Lindsey, 1986). De Wys (1985) has suggested that the increase in blood glucose level seen in cancer patients may delay gastric emptying, resulting in a prolonged sense of fullness and further suppression of appetite. This pattern of elevation of blood glucose may partly explain the reduced appetite for meals other than breakfast, as blood glucose will have had the time to return to normal overnight. There may also, of course, be a psychological component to satiety and anorexia.

Morrison (1984) carried out a study to look at the distribution of food intake in two rat-tumour models. It was found that there was a reduction in both the average size of the meals and frequency of feeding. On the basis of these

Correspondence: M.L. Slevin, Department of Medical Oncology, St Bartholomews Hospital, London EC1 7BE.
Received 8 March 1991; and in revised form 13 November 1991.

data it was hypothesised that early satiety may be a major contributing factor to decreased food intake in cancer patients.

This study has been carried out to test this hypothesis in patients with advanced cancer, attending the Medical Oncology Department at the Homerton Hospital.

### Patients and methods

Sixty-one previously untreated patients with advanced cancer were studied; 32 complained of anorexia, with or without weight loss, and 29 subjectively assessed their appetite to be normal, and acted as controls. Anorexia was defined as a subjective loss of appetite as assessed by the patients themselves. There were a wide variety of tumour types: 36 lung cancer, five mesothelioma, four colon cancer, three adenocarcinoma of unknown origin and 13 others. Those with gastric involvement/mechanical problems were excluded. The distribution of tumour types was similar within the two groups.

In order to assess appetite, and so assess the incidence of early satiety, patients were starved from midnight to 2 p.m. the following day, and hunger, physical emptiness, mood, and mental activity were measured using visual analogue scales (VAS), described by Silverstone & Goodall (1986), who showed these to be a reliable and valid way of subjectively assessing appetite. The scales were filled in at 08.00, 10.00, 12.00 and 14.00, whereafter patients were allowed to eat as much as they wanted. The amount of food consumed was recorded and classified by the investigator as very small, small, medium or large. Fasting blood glucose levels were also measured at 08.00 and 14.00 to ascertain if those patients with anorexia had a raised blood sugar, as this may in part explain the differences in appetite.

### Results

Forty seven men and 14 women were entered into the study. The mean age of the patients studied was 59 years (range = 38–76). Initially, patients were subdivided into a normal appetite (NA) and anorexic (A1) group, according to their own appetite rating. It was apparent that there was a subgroup of patients in the anorexic group, who showed a marked increase in hunger across the study period, but whose food intake at the end of the study was rated as small or very small. The anorexic group was therefore subdivided into an early satiety (ES) group (hunger rating increase across study >75% with final VAS hunger score >6.5), and an anorexic group (A2) with a less marked increase in hunger. Although the hunger rating for the early satiety group at the end of the study was very similar to the normal appetite group, all of the early satiety patients had small or very small food intake, compared with five of the 29 normal appetite patients $P = 0.00004$, Fisher's exact test). In contrast, when those with anorexia (A2) and early satiety are compared; 16/26 of the anorexia (A2) group *versus* 6/6 of the early satiety group had a small or very small food intake ($P = 0.14$ Fishers's exact test).

The respective mean visual analogue ratings (including standard error of the mean (SEM)) are shown for hunger (Figure 1), emptiness (Figure 2), mood (Figure 3) and mental activity (Figure 4) for the patients with NA, A2 and ES. Each of the three groups experienced a significant increase in hunger across the study period ($P<0.001$, for NA, A2 and ES, paired $t$-test).

For the two groups, NA and A1, two-way analysis of variance was used to look at the effect of time and groups on the four VAS parameters. There was a significant difference between the two groups in hunger score ($t = 6.42$, $P<0.001$) and a trend towards increased emptiness in the NA group, which just reached statistical significance ($t = 2.41$, $P = 0.02$). There was no significant difference between the two groups in either mental activity or mood ($t = 0.18$ $P>0.85$, $t = 0.73$

**Figure 1** Time *vs* mean hunger rating. (—□—) normals ($n = 29$); (—◆—) anorexics ($n = 26$); (—■—) early satiety ($n = 6$).

**Figure 2** Time *vs* mean emptiness rating. (—□—) normals ($n = 29$); (—◆—) anorexics ($n = 26$); (—■—) early satiety ($n = 6$).

$P = 0.47$ respectively), and no difference across the study period in these two parameters within the two groups (mood $P = 0.94$, mental activity $P = 0.76$).

These factors were reanalysed, using analysis of variance, considering the patients as three groups, NA, A2 and ES. For hunger there was an effect of group ($P<0.001$) and time ($P<0.001$). From this model, adjusting for time point, the differences between A2 and ES ($t = 2.59$, $P<0.02$) and NA and A2 ($t = 6.97$, $P<.001$) were significant, but the difference between NA and ES was not significant ($t = 1.37$, $P>0.1$). For emptiness and mood the group effect was significant (emptiness $P<0.001$, mood $P<0.001$). In both cases the difference between A2 and ES was significant (emptiness $t = 3.46$, $P<0.001$, Mood $t = 4.15$ $P<0.001$). The difference between NA and ES was highly significant for mood ($t = 3.88$, $P = <0.001$) but was not significant for emptiness ($t = 1.53$, $P>0.1$). There was also a significant difference between NA and A2 for emptiness ($t = 3.38$ $P<0.001$), but not for mood ($t = 0.514$ $P>0.2$). For mental activity there was neither a group ($P = 0.098$) nor a time ($P = 0.76$) effect. However there was a significant difference between those with A2 and ES ($t = 2.17$ $P<0.05$)

Using the Mann-Whitney U Test no differences were detected between those with NA and A2 at either time point (08.00, $P = 0.8$, 14.00, $P = 0.41$) nor between those with NA and ES (08.00, $P = 0.24$, 14.00, $P = 0.4$).

**Figure 3** Time *vs* mean mood rating. (—⊟—) normals ($n$ = 29); (—◆—) anorexics ($n$ = 26); (—■—) early satiety ($n$ = 6).

**Figure 4** Time *vs* mean mental activity rating. (—⊟—) normals ($n$ = 29); (—◆—) anorexics ($n$ = 26); (—■—) early satiety ($n$ = 6).

### Discussion

The VAS scales showed a difference between NA and A1 for hunger and emptiness, but not for mental activity and mood. When the A1 group was subdivided into A2 and ES it is apparent from Figures 1, 2 and 3 that there were differences between those patients with A2 and those with ES in terms of hunger, emptiness, mood and mental activity.

As a group those with ES showed a significant difference in hunger rating from those with NA and anorexia. At the start of the study patients experiencing ES had a hunger rating similar to that of those with anorexia. However they then demonstrated a marked increase in hunger, which reached the level of those with NA. Additionally, those with ES were significantly more empty and miserable and sleepy than those patients classified as anorexic (A2).

In future studies a measurement of psychological status should be included as it would be helpful to know if the patients with ES are more miserable as a result of the increased hunger, emptiness and reduced food intake, or vice versa. In addition, a study which contains larger numbers of patients with ES may provide more information.

The VAS enabled patients with a normal appetite, anorexia and early satiety to be clearly distinguished in three of the four parameters. Early satiety occurred in 10% (6/61) patients. The incidence was lower in this study when compared with the figures from previously published studies (Table I). However these studies used symptom checklists to assess any nutritional problems, rather than using a study design which incorporates the objective experimental assessment of hunger, as in this study. Some of the difference in the incidence of early satiety may be explained by the differing definitions of this complaint: it has been variously described as 'easy filling', 'abdominal fullness', 'fill up quickly' and 'feeling full after having eaten only a little'. Only one of the studies listed (Theologides, 1976) included in their definition that patients have a reduced intake despite having a good appetite. All patients in this study who demonstrated early satiety complained of anorexia; despite this their hunger rating rose markedly when they were starved. This would seem to suggest that these patients' perception of their appetite was based on the amount they ate rather than the hunger that they experienced, a factor confirmed by the small amount of food intake at the end of the study.

In this study the fasting blood glucose levels did not significantly change. This supports the findings of previous investigators (DeWys, 1977; Marks, 1956, 1957), who report that the fasting blood sugars of the cancer patients, anorectic or non-anorectic, did not differ significantly from that of tumour-free controls.

Early satiety is a difficult problem to both detect and treat. There are currently no well recognised treatments and management is usually palliative. Breakfast has been reported to be the best tolerated meal of the day and the patient should be encouraged to make the most of this meal. Welch *et al.* (1985) have reported that, in healthy volunteers, ileal infusions of corn oil emulsions delayed gastric emptying compared with ileal infusion of albumen and saline. They suggest that lipid may interact with ileal receptors to induce early satiety. Rosenbaum *et al.* (1981) suggest that, for this reason, fatty foods should be avoided and replaced by sweet or starchy foods. In addition, small but frequent meals are recommended.

In a small pilot study metoclopramide has been shown to improve delayed gastric emptying, and improve gastrointestinal symptoms experienced by cancer patients (Shivshanker *et al.*, 1984). More recently, cisapride, a non-dopaminolytic motility-enhancing agent, has been licensed for use in Britain to alleviate early satiety. In a study comparing the efficacy of metoclopramide and cisapride on gastric emptying in patients with non-malignant early satiety, it was found that both drugs accelerated the evacuation of the meal in cases of delayed gastric emptying. However, cisapride shortened the initial emptying time lag and was superior to metoclopramide in this respect (Ghigliani *et al.*, 1987). It may be that cisapride and metoclopramide would be useful agents to offer cancer patients suffering this distressing symptom.

The results of this study seem to suggest that early satiety is a major cause of reduced intake in only a small minority of patients with cancer. Our experience suggests that for many of the patients the symptom may not be noticed, especially by the patients themselves, who, in this study, mistook it for loss of appetite. It may be that health care professionals need to ask more specifically about early satiety, as its management may be different from that for other nutritional problems.

**Table I** Previously reported incidence of early satiety

| *Author and year* | *Definition of early satiety* | *Incidence* |
|---|---|---|
| DeWys *et al.*, 1981 | 'Fill up quickly' | 13% (22/169) |
| Fanelli *et al.*, 1986 | None given | 47% (21/45) |
| Grosvenor *et al.*, 1989 | 'Abdominal fullness' | 61% (155/254) |
| Lindsey & Piper, 1985 | 'Feeling full after having eaten only a little' | 20% (2/10) at study start |
| Neilson *et al.*, 1980 | None given | 62% (82/133) |
| Theologides, 1976 | 'Appetite still good with easy filling' | 39% (10/39) |

### References

BRAY, G.A. & CAMPFIELD, L.A. (1975). Metabolic factors in the control of energy stores. *Metabolism*, **24**, 99-117.

DAVIDSON, S., PASSMORE, R., BROCK, J.F. & TRUSWELL, A.S. (1979) *Human Nutrition and Dietetics*. Churchill Livingstone: Edinburgh.

DE WYS, W.D. (1977). Anorexia in cancer patients. *Cancer Res.*, **37**, 2354-2358.

DE WYS, W.D. (1979) Anorexia as a general effect of cancer. *Cancer*, **43**, 2013-2019.

DE WYS, E., COSTA, G. & HENKIN, R. (1981) Clinical parameters related to anorexia. *Cancer Treat. Rep.*, **65** (suppl 5): 49-52.

DE WYS, W.D. (1985) Management of cancer cachexia. *Semin. Oncol.*, **12**, 452-460.

FANELLI, F.R., CANGIANO, C., CECI, F. & others (1986) Plasma tryptophan and anorexia in human cancer. *Eur. J. Cancer Clin. Oncol.*, **22**, 89-95.

GHIGLIANI, M., IANTORNO, G., VAZQUEZ, S. & VARELA, A. (1987) Acute effects of the gastrokinetics cisapride and metochlopramide on the gastric emptying function in patients with the early satiety syndrome. *Acta. Gastroenterol.latinoam.*, **17**, 43-50.

GLICKSMAN, A.S. & RAWSON, R.W. (1956) Diabetes and altered carbohydrate metabolism in patients with cancer. *Cancer*, **9**, 1127-1134.

GRANT, M.M. (1986) Nutritional interventions: increasing oral intake. *Semin. Oncol. Nurs.* **2**, 36-43.

GROSVENOR, M., BULCAVAGE, L. & CHEBLOWSKI, R.T. (1989) Symptoms potentially influencing weight loss in a cancer population: correlations with primary site, nutritional status, and chemotherapy administration. *Cancer*, **63**, 330-334.

HOLROYDE, C.P., GABUZDA, T.G., PUTNAM, R.C., PAUL, P. & REICHARD, G.A., (1975) Altered glucose metabolism in metastatic carcinoma. *Cancer Res.*, **35**, 3710-3714.

HOLROYDE, C.P. & REICHARD, G.A. (1981) Carbohydrate metabolism in cancer cachexia. *Cancer Treat. Rep.*, **65**,(suppl 5): 55-59.

KISNER, D., HAMOSH, M., BELCHER, M. & others (1978). Malignant cachexia: Insulin resistance and insulin receptors. *Proc. Am. Ass. Cancer Res.*, **19**, 199 (Abstract).

KNOX, L.S., CROSBY, L.O., FEURER, I.D., BUZBY, G.P., MILLER, C.L. & MULLEN, J.S. (1983) Energy expenditure in malnourished cancer patients. *Ann. Surg.*, **197**, 152-162.

LINDSEY, A.M. & PIPER, B.F. (1985) Anorexia and weight loss: Indicators of cachexia in small cell lung cancer. *Nutr. Cancer*, **7**, 65-76.

LINDSEY, A., (1986) Cancer cachexia. In *Patholophysiological Phenomena: Human Responses to Illness*. Carreri, V.R.(ed) pp. 122-135. W.B. Saunders: Philadelphia.

LUNDHOLM, K., HOLM, G. & SCHERSTEN, T. (1978) Insulin resistance in patients with cancer. *Cancer Res.*, **38**, 4665-4670.

MARKS, P.A. & BISHOP, J.S. (1957) The glucose metabolism of patients with malignant disease and of normal subjects as studied by means of an intravenous glucose tolerance test. *J. Clin. Invest.*, **36**, 254-257.

MAYER, J. (1953) Glucostatic mechanism of regulation of food intake. *N. Eng. J. Med.*, **249**, 13-16.

MAYER, J. (1955) Regulation of energy intake and body weight: the glucostatic theory and the lipostatic hypothesis. *Ann. N.Y. Aced. Sci.*, **63**, 15-43.

MAYER, J. (1957) Hunger and the hypothalamus. *Clin. Res. Proc.*, **5**, 123-126.

MORRISON, S.D. (1984) Contributions of reduced hunger and premature satiety to cancerous hypophagia in rats. *Cancer Res.*, **44**, 1041-1043.

NEILSON, S.S., THEOLOGIDES, A. & VICKERS, Z.M. (1980) Influence of food odors on food aversions and preferences in patients with cancer. *Am. J. Clin. Nutr.*, **33**, 2253-2261.

SHIVSHANKER, K., BENNETT, R.W. & HAYNIE, T.P. (1983) Tumour associated gastroparesis: Correction with metoclopramide. *Am. J. Surg.*, **145**, 221-225.

SILVERSTONE, T. & GOODALL, E. (1986) Measurement of hunger and food intake. In *Disorders of Eating Behaviour: A Psychoneuroendocrine Approach*. Ferrari, E., Brambilla, F. (eds). Pergamon Press: Oxford. pp. 129-134.

THEOLOGIDES, A. (1972) Pathogenesis of cachexia in cancer: a review and hypothesis. *Cancer*, **29**, 484-488.

THEOLOGIDES, A. (1974). The anorexia-cachexia syndrome: a new hypothesis. *Ann. N.Y. Acad. Sci.*, **230**, 14-22.

THEOLOGIDES, A. (1976) Anorexia-producing intermediary metabolites. *Am. J. Clin. Nutr.*, **29**, 552-558.

THEOLOGIDES, A. (1977) Cancer cachexia in nutrition and cancer. In *Current Concepts in Nutrition*, Winick, M. (ed) **6**, 75-94. John Wiley: New York.

THEOLOGIDES, A. (1979). Cancer Cachexia. *Cancer*, **43**, 2004-2012.

WARBURG, O. (1956) On the origin of cancer cells. *Science*, **123**, 309.

WARREN, S. (1932) The immediate causes of death in cancer. *Am. J. Med. Sci.*, **184**, 610-615.

WATERHOUSE, C. (1974) Lactate metabolism in patients with cancer. *Cancer*, **33**, 66-71.

WELCH, I., SAUNDERS, K. & READ, N.W. (1985) Effect of ileal infusions and intravenous infusions of fat emulsions on feeding and satiety in human volunteers. *Gastroenterol.*, **89**, 1293-1297.

YOUNG, V.R. (1977) Energy metabolism and requirements in the cancer patient. *Cancer Res.*, **37**, 2336-2347.

YOUNG, G.A., COLLINS, J.P. & HILL, G.L. (1979) Plasma proteins in patients receiving intravenous amino acids of intravenous hyperalimentation after major surgery. *Am. J. Clin. Nutr.*, **32**, 92-1199.


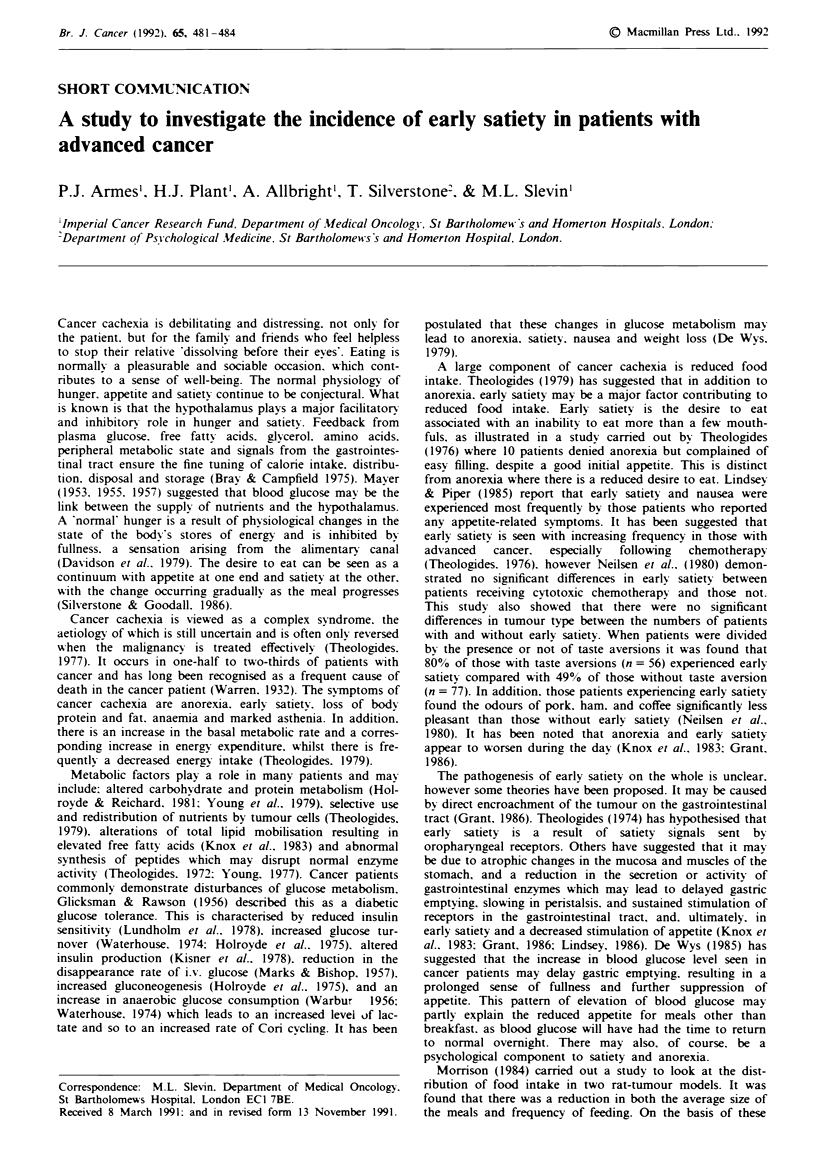

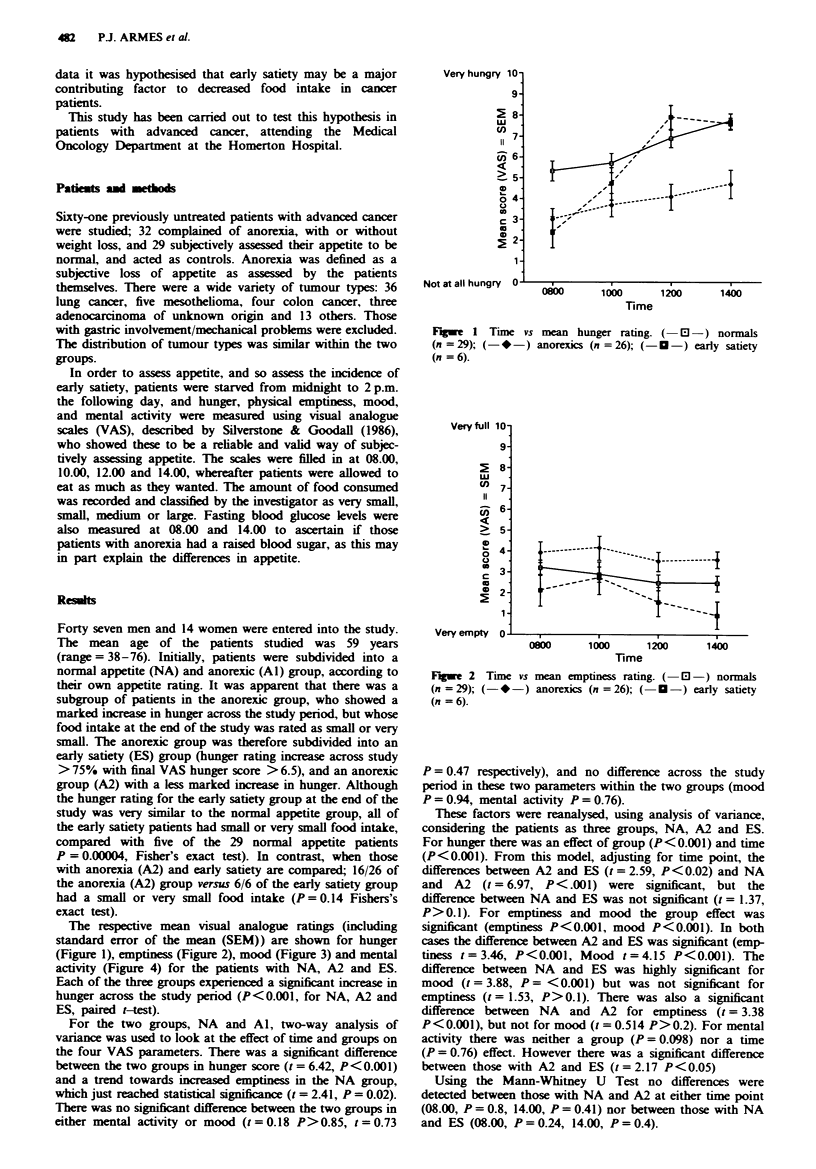

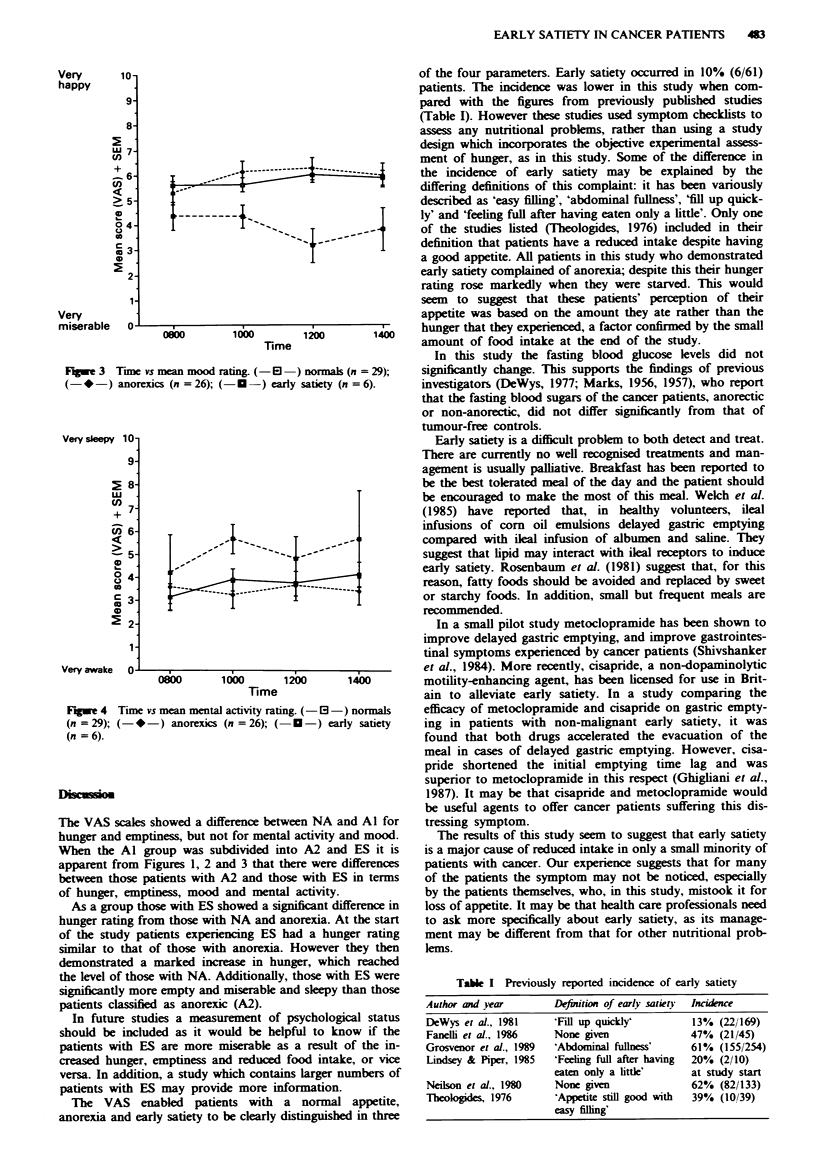

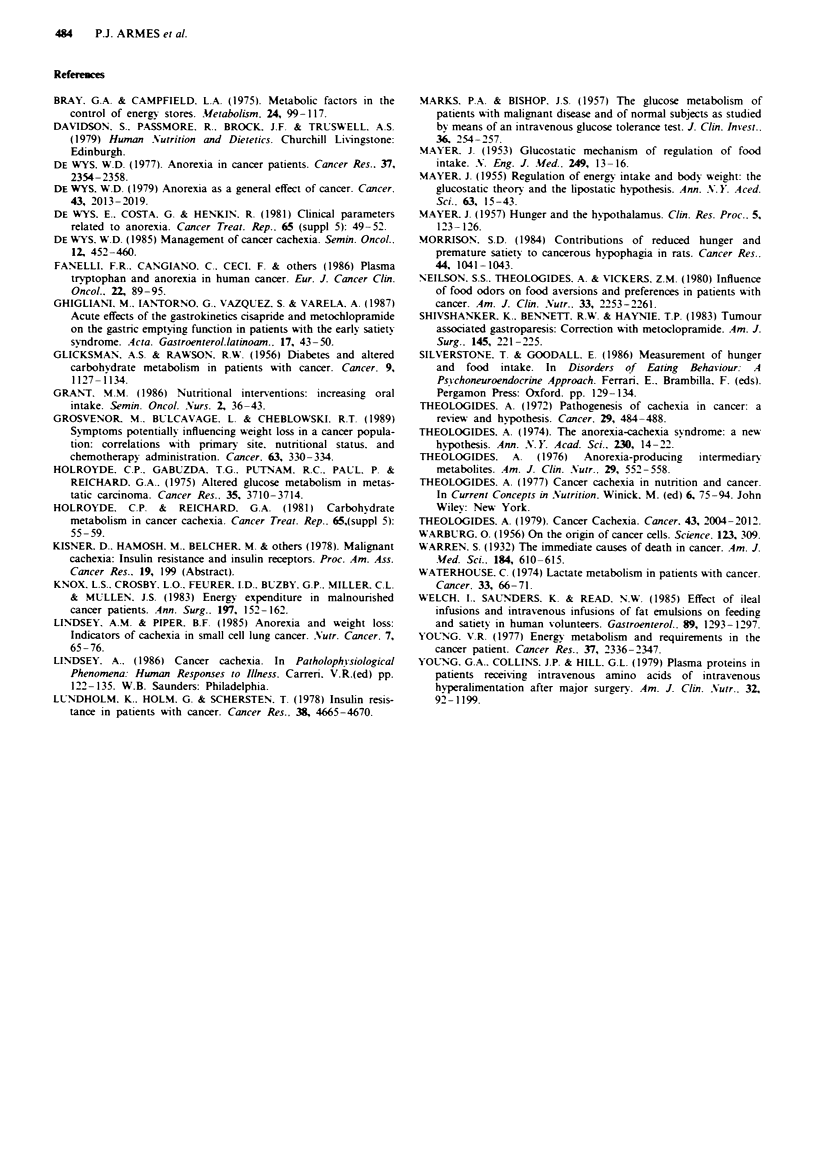


## References

[OCR_00550] Bray G. A., Campfield L. A. (1975). Metabolic factors in the control of energy stores.. Metabolism.

[OCR_00567] DeWys W. D., Costa G., Henkin R. (1981). Clinical parameters related to anorexia.. Cancer Treat Rep.

[OCR_00584] GLICKSMAN A. S., RAWSON R. W. (1956). Diabetes and altered carbohydrate metabolism in patients with cancer.. Cancer.

[OCR_00578] Ghigliani M., Iantorno G., Vázquez S., Varela A. (1987). Acute effect of the gastrokinetics cisapride and metoclopramide on the gastric emptying function in patients with the early satiety syndrome.. Acta Gastroenterol Latinoam.

[OCR_00593] Grosvenor M., Bulcavage L., Chlebowski R. T. (1989). Symptoms potentially influencing weight loss in a cancer population. Correlations with primary site, nutritional status, and chemotherapy administration.. Cancer.

[OCR_00602] Holroyde C. P., Gabuzda T. G., Putnam R. C., Paul P., Reichard G. A. (1975). Altered glucose metabolism in metastatic carcinoma.. Cancer Res.

[OCR_00604] Holroyde C. P., Reichard G. A. (1981). Carbohydrate metabolism in cancer cachexia.. Cancer Treat Rep.

[OCR_00614] Knox L. S., Crosby L. O., Feurer I. D., Buzby G. P., Miller C. L., Mullen J. L. (1983). Energy expenditure in malnourished cancer patients.. Ann Surg.

[OCR_00619] Lindsey A. M., Piper B. F. (1985). Anorexia and weight loss: indicators of cachexia in small cell lung cancer.. Nutr Cancer.

[OCR_00629] Lundholm K., Holm G., Scherstén T. (1978). Insulin resistance in patients with cancer.. Cancer Res.

[OCR_00635] MARKS P. A., BISHOP J. S. (1957). The glucose metabolism of patients with malignant disease and of normal subjects as studied by means of an intravenous glucose tolerance test.. J Clin Invest.

[OCR_00641] MAYER J. (1953). Glucostatic mechanism of regulation of food intake.. N Engl J Med.

[OCR_00643] MAYER J. (1955). Regulation of energy intake and the body weight: the glucostatic theory and the lipostatic hypothesis.. Ann N Y Acad Sci.

[OCR_00652] Morrison S. D. (1984). Contributions of reduced hunger and premature satiety to cancerous hypophagia in rats.. Cancer Res.

[OCR_00657] Nielsen S. S., Theologides A., Vickers Z. M. (1980). Influence of food odors on food aversions and preferences in patients with cancer.. Am J Clin Nutr.

[OCR_00573] Rossi Fanelli F., Cangiano C., Ceci F., Cellerino R., Franchi F., Menichetti E. T., Muscaritoli M., Cascino A. (1986). Plasma tryptophan and anorexia in human cancer.. Eur J Cancer Clin Oncol.

[OCR_00662] Shivshanker K., Bennett R. W., Haynie T. P. (1983). Tumor-associated gastroparesis: correction with metoclopramide.. Am J Surg.

[OCR_00681] Theologides A. (1976). Anorexia-producing intermediary metabolites.. Am J Clin Nutr.

[OCR_00690] Theologides A. (1979). Cancer cachexia.. Cancer.

[OCR_00685] Theologides A. (1977). Cancer cachexia.. Curr Concepts Nutr.

[OCR_00679] Theologides A. (1974). Generalized perturbations in host physiology caused by localized tumors. The anorexia-cachexia syndrome: a new hypothesis.. Ann N Y Acad Sci.

[OCR_00673] Theologides A. (1972). Pathogenesis of cachexia in cancer. A review and a hypothesis.. Cancer.

[OCR_00691] WARBURG O. (1956). On the origin of cancer cells.. Science.

[OCR_00696] Waterhouse C. (1974). Lactate metabolism in patients with cancer.. Cancer.

[OCR_00700] Welch I., Saunders K., Read N. W. (1985). Effect of ileal and intravenous infusions of fat emulsions on feeding and satiety in human volunteers.. Gastroenterology.

[OCR_00708] Young G. A., Collins J. P., Hill G. L. (1979). Plasma proteins in patients receiving intravenous amino acids or intravenous hyperalimentation after major surgery.. Am J Clin Nutr.

